# The Effect of Sex on the Risk of Long-COVID and Cardiovascular Complications in Healthy Patients without Comorbidities: Data from a Polish Long-COVID Cardiovascular (PoLoCOV-CVD) Study

**DOI:** 10.3390/jcm13061559

**Published:** 2024-03-08

**Authors:** Agata Bielecka-Dabrowa, Agata Sakowicz, Katarzyna Gryglewska-Wawrzak, Joanna Kapusta, Maciej Banach, Piotr Jankowski, Michał Chudzik

**Affiliations:** 1 Department of Preventive Cardiology and Lipidology, Medical University of Lodz, 90-419 Lodz, Poland; maciej.banach@icloud.com; 2 Department of Cardiology and Congenital Diseases of Adults, Polish Mother’s Memorial Hospital Research Institute (PMMHRI), 93-338 Lodz, Poland; 3 Department of Medical Biotechnology, Medical University of Lodz, 90-419 Lodz, Poland; agata.sakowicz@gmail.com; 4 Department of Internal Diseases, Rehabilitation and Physical Medicine, Medical University of Lodz, 90-419 Lodz, Poland; joanna.kapusta@umed.lodz.pl; 5 Ciccarone Center for the Prevention of Cardiovascular Disease, School of Medicine, Johns Hopkins University, Baltimore, MD 21205, USA; 6 Department of Internal Medicine and Geriatric Cardiology, Centre of Postgraduate Medical Education, 01-813 Warsaw, Poland; piotrjankowski@interia.pl (P.J.); michalchudzik@wp.pl (M.C.); 7 Department of Epidemiology and Health Promotion, School of Public Health, Center of Postgraduate Medical Education, 01-813 Warsaw, Poland; 8 Department of Nephrology, Hypertension and Family Medicine, Medical University of Lodz, 92-213 Lodz, Poland

**Keywords:** COVID-19, COVID complications, long-COVID, women’s health, sex differences

## Abstract

**Background:** The prevalence of long-COVID (LC) presents a significant challenge to healthcare systems globally. There are still some discrepancies on the role of sex as an independent risk factor of LC complications. Thus, we aimed to determine the differences in clinical and cardiovascular complications between males and females without comorbidities after COVID-19. **Methods:** Clinical data on the course of the disease with the accompanying symptoms and post-COVID-19 symptoms were compiled from both male and female subjects with a minimum 12-week interval after COVID-19 recovery. Next, the patients were followed for 12 months. ECG, echocardiography, 24 h ECG monitoring, 24 h ambulatory blood pressure monitoring (ABPM), and selected biochemical tests were performed. LC was diagnosed based on the World Health Organization (WHO) definition. To reduce the impact of confounders, i.e., body mass index (BMI) and age, on the results of the study, the nearest neighbour (NN) propensity score matching (PSM) method with a 1:1 ratio was used. **Results**: The results were obtained following the removal of cases with comorbidities from the database consisting of 1237 males and 2192 females, and PSM of the new database included 886 cases (443 males and 443 females). At both the 3-month and 1-year post-recovery marks, females consistently reported a higher frequency of LC symptoms compared to males (*p* < 0.001 for both comparisons). Moreover, after 1 year of follow-up, females exhibited a higher prevalence of LC compared to males, with rates of 14% versus 8.3%, respectively (*p* = 0.013). The symptoms that significantly differed between females and males in the 12-month follow-up were hair loss (5.4 vs. 0.7%, *p* < 0.001), memory and concentration disturbances (8.4 vs. 4.3%, *p* = 0.013), and headaches (4.3 vs. 1.4%, *p* = 0.008). Females presented lower mean arterial pressure (MAP) [89 (83–95) mmHg versus (vs.) 94 (89–100); *p* < 0.001] and lower pulse pressure (PP) [46 (42–52) mmHg vs. 51 (48–57); *p* < 0.001] in 24 h ABPM and more elevated heart rates (HRs) in 24 h ECG monitoring as well as arrhythmia (*p* < 0.001 and *p* = 0.018, respectively). Males had a higher occurrence of ECG abnormalities such as QRS >= 120 ms, ST-T changes, T inversion, arrhythmia, and QRS fragmentation (27.3% vs. 19.2%; *p* = 0.004). No significant differences were observed between males and females concerning physical activity levels, stress, fatigue, alcohol consumption, and smoking habits. **Conclusions:** One year post-COVID-19 recovery, regardless of age and BMI, healthy females more often suffered from LC symptoms than males. They had lower MAP and PP in 24 h ABPM, more often had higher HRs and arrhythmia in 24 h ECG monitoring, and fewer ECG abnormalities than males.

## 1. Introduction

COVID-19 is a severe respiratory syndrome caused by coronavirus 2 (SARS-CoV-2). In December 2019, it was first identified in Wuhan, China, and has since become a global epidemic [1]. The symptoms of COVID-19 vary in severity and can range from mild flu-like symptoms to severe respiratory distress [2]. In some cases, the infection can lead to severe pneumonia, multiorgan failure, and even death, particularly in vulnerable populations such as older adults and those with underlying health conditions [3]. To mitigate the spread of the virus, public health measures such as wearing face masks, practicing physical distancing, frequent handwashing, and avoiding large gatherings have been recommended [4,5]. Vaccines have also been developed and are being administered worldwide to prevent COVID-19 and reduce its impact [6]. The pandemic has had significant social, economic, and healthcare consequences. One of them is long-COVID (LC) syndrome, also known as post-acute sequelae of SARS-CoV-2 infection, which refers to a condition where individuals continue to experience a range of symptoms and health issues for weeks or even months after initially contracting COVID-19 [7]. The symptoms of long-COVID might be diverse: some common manifestations include fatigue, exercise intolerance, shortness of breath, cough, chest pain, joint and muscle pain, headaches, brain fog, difficulty concentrating, memory problems, insomnia, loss of taste and smell, dizziness, anxiety, and depression, to serious cardiovascular complications like myocarditis or acute coronary syndrome [8,9,10,11,12]. The exact mechanisms underlying long-COVID are not yet fully understood. Some theories suggest that it could be related to persistent viral activity, immune dysfunction, inflammation, or organ and tissue damage caused by the initial infection [13]. Long-COVID can affect individuals of all ages, including those who initially had mild or asymptomatic cases initially [9,14]. Since long-COVID is a relatively new phenomenon, research is ongoing to better understand its long-term implications and develop effective interventions. Research on the sex influence in developing long-COVID is still evolving, and the available data are not yet conclusive. However, some studies have suggested that there may be differences in the prevalence and severity of long-COVID symptoms between sexes [15,16]. Therefore, the aim of the study was to assess age-independent differences between males and females without comorbidities on the risk of long-COVID and long-COVID clinical and cardiovascular complications [17].

## 2. Materials and Methods

### 2.1. Basic Characteristics

Following the removal of the cases with comorbidities from the database consisting of 1237 males and 2192 females, and the propensity score matching (PSM) using the neighbour method with the 1:1 ratio, the new database included 886 cases [443 males (mean age 44) and 443 females (mean age 43)]. All subjects completed the 12-month follow-up. The patients included in the Polish Long-COVID Cardiovascular (PoLoCOV-CVD) Study are part of the STOP-COVID registry (ClinicalTrials.gov identifier—NCT05018052). The details of the study and the registry have been thoroughly elucidated previously [18]. In short, the Polish Long COVID Cardiovascular Study (PoLoCOV-CVD) is a prospective non-intervention study carried out among patients in ambulatory primary care in Poland. The Bioethics Committee of Lodz Regional Medical Chamber (K.B.-0115/2021) approved the study. 

Patients from selected centres took part in our registry, mainly from Lodz and Warsaw. We included patients meeting the inclusion criteria: (1) age ≥ 18 years; (2) confirmed diagnosis of COVID-19, in accordance with the current guidelines of the European Centre for Disease Prevention and Control [a confirmed case is a person who meets at least one of the following laboratory criteria: SARS-CoV-2 nucleic acid detection; SARS-CoV-2 antigen identification in clinical specimens (excluding self-tests carried out outside the health care environment; or SARS-CoV-2 isolation in clinical specimens); and (3) complete recovery (resolution of acute clinical symptoms, minimum of 14 days after the last symptoms)]. All diseases, except obesity, are considered a criterion of exclusion. There was the possibility of undergoing Holter medical assessment and echocardiographic tests for patients after COVID-19, regardless of their clinical symptoms. Based on data from the Polish Long-COVID Cardiovascular (PoLoCOV-CVD) Study, we evaluated the LC predictor in healthy people over the age of 18, without comorbidity, diagnosed with COVID-19 and after full recovery (resolution of clinical symptoms, minimum of 14 days after last symptoms) regardless of hospitalization. Long-COVID was recognised on the basis of the WHO-approved definition [19]. LC was identified when, after three months of COVID-19, new symptoms that appeared during the acute period remained and/or were aggravated. Symptoms of COVID-19 were assessed during the patient’s interview at the subsequent visit. All subjects included in the study were informed in detail about the research and gave their written consent to participate in the study [18]. Patients’ data, including disease progression, post-COVID-19 symptoms, and underlying health conditions, were gathered during the initial visit (“visit 0”), which occurred within 12 weeks after the end of COVID-19. All participants completed the 12-month follow-up. In outpatient clinics, all patients were thoroughly screened and physically examined. The collected information included the existence or absence of COVID-19 symptoms, general symptoms, and their characteristics, if any. Furthermore, all patients received the following medical tests: (1) 12-lead electrocardiogram (ECG) (BTL Industries Limited, Warsaw, Poland); (2) 24 h Holter ECG monitoring (Medicalgorithmics, Warsaw, Poland); (3) 24 h ambulatory blood pressure monitoring (ABPM) (BTL Industries Limited, Warsaw, Poland); (4) echocardiography (GE Healthcare, Chicago, IL, USA), within which quantitative measures were obtained in accordance with the guidelines of the European Society of Cardiology (ESC)—the left ventricular (LV) volumes and ejection fraction (EF) were derived according to the modified biplane Simpson’s rule and right ventricular (RV) functional measures were tricuspid annular plane systolic excursion (TAPSE); (5) cardiac magnetic resonance (MRI) (Philips, Eindhoven, The Netherlands); and (6) biochemical tests: lipidogram [total cholesterol, triglycerides, LDL (low-density lipoprotein) cholesterol, HDL (high-density lipoprotein) cholesterol)] [18].

### 2.2. Statistical Analysis

**Figure 1:**
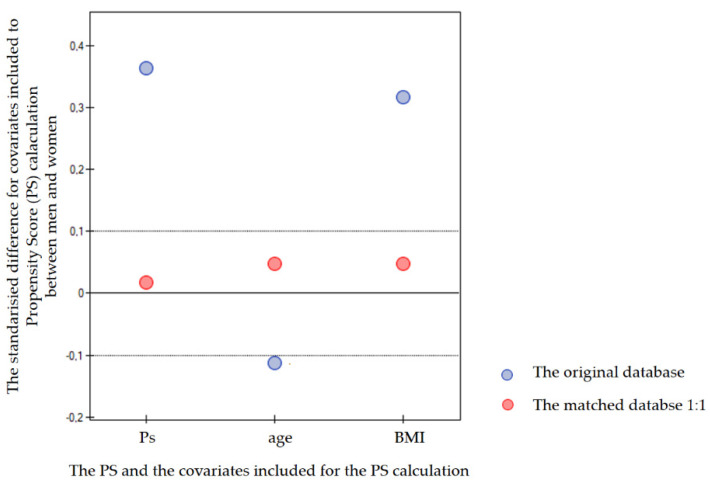
The standardised difference between males and females for the mean of covariates included in the propensity score (PS) calculation as well as between the mean propensity scores.

The statistical analyses were conducted using the following tools: Statistica 13.1 (StatSoft, Cracow, Poland) and PQStat 1.8.4 (PQStat software, Poznan, Poland). The distribution of continuous data was determined using the Shapiro–Wilk test. As all-continuous data presented a non-normal distribution, they were analysed using a U Mann–Whitney test. The categorical data were examined using chi2 or chi2 with Yates correction or a Fisher test. The logistic regression model was used to calculate the propensity score for each subject in the database. The propensity score matching (PSM) was conducted using the neighbour method with a 1:1 ratio. A *p*-value below 0.05 was considered significant. We used propensity score matching (PSM). From the database consisting of 1237 males and 2192 females, we eliminated cases of those with the comorbidities, and significant age differences occur. Next, the logistic regression model for the calculation of the individual propensity scores for each patient was used. To minimize the selection bias in age and BMI between men and women, the men were matched to women using the PSM method. Following the propensity score matching (PSM) using the neighbours’ method with a 1:1 ratio, we obtained a database consisting of 886 cases, i.e., 443 males and 443 females. The standardised differences between the calculated propensity scores for BMI and age, as well as for both, i.e., BMI + age, before and after matching are presented in Figure 1. 

## 3. Results

### 3.1. Main Characteristics

Finally, after PSM, our database included 886 subjects—443 males and 443 females. During COVID-19, males required more frequent hospitalisation for pneumonia (*p* < 0.001). However, there was no difference in their stay in Intensive Care Units (ICUs). Females reported a trivial, moderate, and severe course of infection more frequently than males (*p* = 0.015). There were no differences in sport activity, level of stress and fatigue, as well as alcohol drinking and smoking between males and females. Data are presented in Table 1. 

Symptoms in the 12-month follow-up were more common in females (14% vs. 8.3%; *p* = 0.013) compared to males. The symptoms that significantly differed between females and males 3 months after COVID-19 recovery were fatigue (28 in females vs. 20% in males; *p* = 0.005), hair loss (7 vs. 0.3%; *p* < 0.001) and memory and concentration disturbances [“brain fog”—informal term for a common complaint of intellectual functions among patients with post-acute COVID-19] (12 vs. 7%; *p* = 0.013). In the one year follow-up, females reported more common hair loss (5.4 vs. 0.7%, *p* < 0.001), memory and concentration disturbances (8.4 vs. 4.3%, *p* = 0.013), and headaches (4.3 vs. 1.4%, *p* = 0.008). The number of symptoms was significantly greater in females than in males 3 months and one year after COVID-19 [median 5 (range: 2–7) vs. median 3 (range: 2–6), *p* < 0.001; median 2 (range: 0–5) vs. median 0 (range: 0–2), *p* < 0.001; respectively]. Results are presented in Table 2.

### 3.2. Differences between Groups 12 Months after COVID-19 Recovery

Males had more ECG abnormalities (any abnormality: heart rate > 100/min; QRS ≥ 120 ms; ST-T changes; arrhythmia; fragmentation of QRS complex) than females (27.3 in males vs. 19.2% in females; *p* = 0.004). There were no significant differences in myocardial damage evaluated in late gadolinium enhancement cardiac magnetic resonance (CMR) between sexes. Considering echocardiography, left atrial (LA), aortic (AD), and right ventricle (RV) diameters were greater in males in comparison to females (*p* < 0.001, for all). On the other hand, females presented more elevated heart rates (HRs) in 24 h ECG monitoring as well as arrythmia than males (*p* < 0.001 and *p* = 0.018, respectively). Females presented with significantly higher levels of HDL, but lower level of triglycerides (TG) and non-HDL compared to males (*p* < 0.001, for all). Regarding 24 h ABPM assessment, the mean arterial pressure (MAP) and pulse pressure (PP) were lower in females (*p* < 0.001, for all). Detailed results are presented in Table 3.

## 4. Discussion

This study aimed to investigate the differences in long-term symptoms between females and males who had recovered from COVID-19, without comorbidities. During the acute phase of COVID-19, the study found that males had a higher incidence of hospitalization due to pneumonia compared to females (12.5% vs. 5.5%). However, there were no significant differences in ICU stays between the two sexes. Pneumonia in COVID-19 is a serious and life-threatening respiratory complication caused by the SARS-CoV-2 virus. Common symptoms of COVID-19 pneumonia include fever, cough, shortness of breath, and difficulty breathing. In severe cases, patients may experience acute respiratory distress syndrome (ARDS), a condition characterized by severe lung inflammation and respiratory failure [20,21]. Multiple investigations have underscored a propensity for male individuals to manifest more symptomatic trajectories of COVID-19 when juxtaposed with their female counterparts. Notably, in the meta-analysis conducted by Abate et al., comprising 57 studies encompassing 221,195 participants, a discernible trend was observed: the prevalence of symptomatic COVID-19 cases was notably elevated among males in comparison to females [22]. In the findings of another study on 224 COVID-19 patients requiring mechanical ventilation, the male patients displayed a higher severity of COVID-19. This was reflected in higher rates of vasopressors, duration of stay, and duration of intubation. On the other hand, no significant differences were observed in mortality rates, organ replacement therapy, and complications during ICU stay [23].

In our findings, females reported a higher number of long-COVID symptoms compared to “matching” males, both at 3 months and after one year. Furthermore, after one year, a higher percentage of females experienced long-term COVID symptoms compared to males. The suggestion of female sex as a potential risk factor for developing long-COVID symptoms was made in earlier research [24,25]. Furthermore, some multicenter studies confirmed these results [26,27,28]. The specific long-COVID symptoms that differed significantly between females and males in the current study were memory and concentration disturbances, hair loss, and headaches, which were more prevalent in females. Cognitive impairments, often referred to as “brain fog” can also have significant effects on various aspects of daily life [29]. COVID-19 has the potential to impact cognitive function by engaging mechanisms like infecting neurons, activating microglia, and damaging vascular endothelial cells [30]. Lam et al. investigated long-COVID brain fog and found that, at 8 months post-infection, females were independently associated with subjective neurocognitive impairment [31]. The exact mechanisms that cause hair loss in long-COVID are not fully understood. Telogen effluvium (TE) is one of the most popular alopecies caused by physical and emotional stress, hormonal imbalances, inflammation, and nutritional deficiencies [32,33,34]. In the study of Olds et al. the authors documented 10 cases of TE post-COVID-19 infection, with most affected individuals being female [35]. In a meta-analysis which included 465 patients with acute TE (67.5% females), the most common trichoscopic findings were decreased hair density, the presence of empty follicles, or short regrowing hair [36]. Headaches are commonly reported by individuals with long-COVID, causing considerable discomfort and influencing their overall quality of life [37]. One meta-analysis found that the prevalence of post-COVID headache ranged from 8% to 15% during the first 6 months after SARS-CoV-2 infection [38]. In the study by Michelutti et al., a higher prevalence of headaches was observed among females (14.6% vs. 3.2%; *p* = 0.080) [39].

In our next findings, females demonstrated lower MAP and PP in 24 h ABPM compared to males after one year of recovery from COVID-19. Recent evidence indicates that COVID-19 could lead to hypertension [40,41]. Several studies have observed elevated blood pressure levels and an increased prevalence of hypertension as post-acute sequelae of COVID-19 [42,43]. MAP refers to the average arterial pressure during a complete cardiac cycle, systole, and diastole phases [44]. Kundu et al. proposed that MAP is a more effective approach for predicting blood pressure compared to using systolic blood pressure (SBP) or diastolic blood pressure (DBP) separately. The utility of using MAP was demonstrated by investigating the relationship between blood pressure and possible causal factors, and in improving the capability of identifying mild cases of hypertension [45]. PP is the difference between systolic and diastolic blood pressure [46]. The high value of PP (≥60 mmHg) is associated with certain cardiovascular conditions [47]. In the study of Takegami et al., home blood pressure was monitored over a continuous period of 14 days in a group of 1082 patients diagnosed with type 2 diabetes. Subsequently, PP was calculated. The risk of cardiovascular disease was 1.88 times higher in the morning in the higher PP group than in the lower PP group [48]. In another study, the authors investigated the relationship between PP and all-cause mortality in 1581 ischaemic heart failure (HF) patients with left ventricular systolic dysfunction (LVSD). A J-shaped relationship between PP and all-cause mortality was observed in ischaemic HF patients with LVSD, and higher PP was associated with a worse prognosis only in those with SBP ≥110 mmHg [49].

Studies have indicated that there are variations in pulse pressure between males and females. Skurnick et al. undertook a meta-analysis elucidating that females exhibit lower PP levels than males during early adulthood, with a contrasting trend emerging in older age cohorts where females demonstrate higher PP levels. Moreover, the study identified a distinct pattern: females manifest a steeper, consistent escalation in PP with advancing age, a statistically significant observation (*p* < 0.001). Conversely, males exhibit a more pronounced curvilinear rise in PP with increasing age (*p* = 0.006) [50]. Another study has indicated that PP amplification serves as a strong predictor of variations in cardiovascular (CV) risk between males and females. In post-menopausal females, the attenuation of PP amplification, mainly related to increased aortic stiffness, contributes to the significant increase in CV risk [51]. Kang et al. demonstrated that increases in forward wave pressure (Pf), backward wave pressure (Pb), and DBP are relevant factors that contribute to the augmented aortic PP response to postexercise muscle ischemia [52].

In the presented study, females demonstrated a more elevated heart rate and arrhythmia in 24 h ECG monitoring. On the other hand, males exhibited a higher incidence of certain ECG abnormalities, including QRS duration that is greater than or equal to 120 ms, ST-T changes, T wave inversions, arrhythmias, and QRS fragmentation compared to females. In some studies, long-COVID patients have been observed to experience temporary or ongoing ECG and Holter-ECG abnormalities, with frequencies varying from less than 1% in young athletes to as high as 27.5% in hospitalized patients with cardiovascular complications [12,53]. Considering the findings of our study, it appears beneficial to conduct Holter-ECG monitoring more frequently in patients following COVID-19, particularly in women. Based on the results of ECG monitoring and the observed tendency towards higher heart rates after COVID-19, females may require more frequent treatment with beta-blockers.

In our study, we observed differences between males and females in echocardiography parameters. It is noteworthy that sex differences in echocardiographic measures may exist independently of long-COVID, such as LV linear dimensions and left atrial volume, and can be explained on the grounds of the smaller body sizes of women [54].

Our analysis focuses on a specific population of individuals without comorbidities, which helps to isolate the impact of long-COVID on otherwise healthy individuals. This targeted approach enhances the internal validity of the findings and provides valuable insights into the potential risks and complications in this specific subgroup. Moreover, the study employed a prospective design, following participants over a one-year period, which allows for the examination of long-term outcomes and the assessment of symptom persistence over time.

Our analysis has some obvious limitations and strengths. The study was limited to primary care interventions. Only selected biochemical parameters were evaluated in the patients we examined. We have no detailed information about MRI protocols. We used self-reported data to evaluate persistent COVID-19 symptoms. Patients in our study have not been examined for respiratory function, and the exact degree of functional decline is unknown. Instead, they only compare their perceptions to their previous respiratory functions from a subjective point of view. However, diagnoses are carefully made to minimize potential confounders such as pre-existing symptoms, simultaneous infections from other viral agents, and underlying comorbidities. We have no healthy control without infection of COVID-19 in our study. Despite these limitations, the study makes significant contributions to our understanding of long-COVID and its impact on both sexes, providing valuable information on potential cardiovascular implications and the persistence of symptoms over time. Further research is required to fully comprehend the underlying mechanisms driving these disparities and to validate the study findings.

## 5. Conclusions

In conclusion, females one year after COVID-19 recovery without comorbidities more often suffered from long-COVID than males regardless of age and BMI. Females after COVID-19 had lower mean arterial pressure and pulse pressure in 24 h ABPM, more often higher heart rates and arrhythmia in 24 h ECG monitoring, and fewer ECG abnormalities than males.

## Figures and Tables

**Table 1:** The physical description of the basic populations and after PSM.

Variables	Before Matching			After Matching		
	Females *n* = 2192	Males *n* = 1237	*p*	Females *n* = 443	Males *n* = 443	*p*
Age	53 (43–63)	52 (42–63)	0.203	43 (37–52)	44 (35–54)	0.495
BMI	26.4 (23.1–30.5)	28.4 (25.6–31.6)	<0.001	26.2 (23.1–29.8)	26.8 (24.4–29.6)	0.117
COVID-19 vaccination	1821 (83%)	1043 (84%)	0.346	63 (14%)	53 (12%)	0.320
**Lifestyle**						
No smoking and alcohol	1975 (90%)	1044 (84%)	<0.001	383 (87%)	375 (85%)	0.610
Smoking	190 (9%)	104 (8%)		42 (9.5%)	44 (9.9%)	
Alcohol	27 (1%)	86 (7%)		18 (4.1%)	24 (5.4%)	
Stress/fatigue/overwork	826 (38%)	371 (30%)	<0.001	105 (24%)	105 (24%)	0.571
Regular physical activity	622 (28%)	422 (34%)	<0.001	105 (24%)	109 (25%)	0.754
**The course of COVID infection**						
Home isolation	1905 (87%)	953 (77%)	<0.001	382 (86%)	367 (83%)	0.203
Pneumonia: hospitalisation	197 (9%)	224 (18%)	<0.001	26 (6%)	56 (13%)	<0.001
Hospitalisation in ICU	17 (1%)	14 (1%)	0.289	4 (1%)	3 (0.7%)	1.000
**The strength of COVID infection—subjective assessment of the patient**						
Trivial	151 (7%)	87 (7%)	<0.001	53 (12%)	36 (8.1%)	0.015
Mild	623 (28%)	407 (33%)		137 (31%)	178 (40%)	
Moderate	720 (33%)	291 (24%)		124 (28%)	104 (23%)	
Severe	699 (32%)	453 (37%)		129 (29%)	125 (28%)	

Abbreviations: BMI—body mass index; ICU—intensive care unit. Definitions: The strength of COVID infection—subjective assessment of the patient: trivial—home course with symptoms < 14 days, subjective evaluation by the patient as severe (“1” on a scale of 1–3); mild—home course with symptoms > 14 days, subjective evaluation by the patient as severe (“1” on a scale of 1–3); moderate—home course with symptoms lasting > 14 days, subjective evaluation by the patient as severe (“2” on a scale of 1–3); severe—one of the following: home course with symptoms lasting > 14 days, subjective evaluation by the patient as severe (“3” on a scale of 1–3), with temperature > 38 °C, dyspnea, or saturation below 94 lasting more than 3 days; hospitalization with diagnosis: pneumonia, respiratory failure, intensive care unit, assisted breathing, thromboembolic complications during hospitalization.

**Table 2:** Long-COVID symptoms 3 months and one year after COVID-19 recovery.

Variables	Females (*n* = 443)	Males (*n* = 443)	*p*
Symptoms (one year after COVID-19)	60 (14%)	37 (8.3%)	0.013
Fatigue (one year after COVID-19)	26 (5.9%)	21 (4.74%)	0.453
Fatigue (3 months after COVID-19)	126 (28%)	90 (20%)	0.005
Dyspnea (one year after COVID-19)	12 (2.7%)	7 (1.6%)	0.246
Dyspnea (3 months after COVID-19)	17 (4%)	14 (3%)	0.583
Dysosmia and dysgeusia (one year after COVID-19)	8 (1.8)	3 (0.7%)	0.129
Dysosmia and dysgeusia (3 months after COVID-19)	24 (5%)	20 (4.5%)	0.536
Musculoskeletal pain (one year after COVID-19)	10 (2.3%)	10 (2.3%)	1.000
Musculoskeletal pain (3 months after COVID-19)	13 (2.7%)	12 (2.9%)	0.839
Hair loss (one year after COVID-19)	24 (5.4%)	3 (0.7%)	<0.001
Hair loss (3 months after COVID-19)	30 (7%)	1 (0.3%)	<0.001
Memory and concentration disturbances (one year after COVID-19)	37 (8.4%)	19 (4.3%)	0.013
Memory and concentration disturbances (3 months after COVID-19)	54 (12%)	32 (7%)	0.013
Sleep disorders, neurosis, depression (one year after COVID-19)	0 (0%)	1 (0.2%)	1.000
Sleep disorders, neurosis, depression (3 months after COVID-19)	2 (0.5%)	2 (0.5%)	1.000
Headache (one year after COVID-19)	19 (4.3%)	6 (1.4%)	0.008
Headache (3 months after COVID-19)	13 (2.9%)	8 (1.8%)	0.269
Sum of symptoms (3 months after COVID-19)	5 (2–7)	3 (2–6)	<0.001
Sum of symptoms (12 months after COVID-19)	2 (0–5)	0 (0–2)	<0.001

Definitions: Memory and concentration disturbances—“brain fog”—informal term for a common complaint of intellectual functions among patients with post-acute COVID-19; sleep disorders—problems with the quality, timing, and amount of sleep, which result in daytime distress and impairment in functioning; neurosis—any one of a variety of mental disorders characterized by significant anxiety or other distressing emotional symptoms, such as persistent and irrational fears, obsessive thoughts, compulsive acts, dissociative states, and somatic and depressive reactions; depression—mental disorder causes severe symptoms that affect how a person feels, thinks, and handles daily activities, such as sleeping, eating, or working. To be diagnosed with depression, the symptoms must be present for at least 2 weeks.

**Table 3:** Differences between investigated groups 12 months after COVID-19 recovery (and after PSM).

Variables	Before Matching			After Matching		
	Females *n* = 2192	Males *n* = 1237	*p*	Females (*n* = 443)	Males (*n* = 443)	*p*
**ECG**						
ECG abnormalities (any abnormality: heart rate > 100/min; QRS ≥ 120 ms; ST-T changes; arrhythmia; fragmentation of QRS complex)	58 (2.6%)	72 (5.8%)	<0.001	85 (19.2%)	121 (27.3%)	0.004
**Echocardiography**						
LVEF (%)	61 (56–67)	60 (56–65)	0.122	60 (56–66)	59 (54–65)	0.249
LA (mm)	37 (35–40)	42 (38–45)	<0.001	36 (34–39)	40 (36–43)	<0.001
AD (mm)	30 (25–32)	33 (30–36)	<0.001	29 (27–31)	32 (30–34)	<0.001
RV (mm)	28 (26–29)	30 (28–32)	<0.001	30 (25–32)	33 (30–35)	<0.001
TAPSE (mm)	25 (24–26)	25 (23–26)	<0.001	25 (24–26)	25 (24–26)	0.536
**Cardiac MRI**						
LGE	106 (4.8%)	86 (7%)	0.010	25 (5.6%)	36 (8.1%)	0.144
Segmental wall-motion abnormalities of the left ventricle	551 (25%)	339 (27%)	0.146	7 (1.6%)	5 (1.1%)	0.561
**24 h ECG ambulatory monitoring**						
Mean HR	75 (69–81)	73 (67–80)	<0.001	77 (69–85)	74 (66–82)	<0.001
Arrhythmia (supraventricular and ventricular extrasystoles)	493 (22.5%)	364 (29.4)	<0.001	40 (9%)	22 (5%)	0.018
**ABPM**						
MAP mean daily	90 (84–96)	94 (88–100)	<0.001	89 (83–95)	94 (89–100)	<0.001
PP mean daily	49 (43–56)	53 (48–58)	<0.001	46 (42–52)	51 (48–57)	<0.001
Systolic dipping	13 (8–18)	12 (7–17)	0.013	13 (9–17)	13 (9–18)	0.831
**Biochemical parameters**						
TC (mg/dL)	196 (169–223)	188 (159–271)	<0.001	192 (169–217)	193 (169–222)	0.456
HDL (mg/dL)	55 (46–64)	60 (51–67)	<0.001	59 (51–66)	49 (44–59)	<0.001
TG (mg/dL)	96 (71–134)	120 (84–168)	<0.001	89 (65–122)	109 (77–159)	<0.001
Non-HDL (mg/dL)	135 (110–162)	137 (108–167)	0.654	130 (110–155)	143 (118–169)	<0.001

Abbreviations: ECG—electrocardiogram; ECG abnormalities, i.e., QRS fragmentation ≥120 ms, ST-T changes, T inversion, arrhythmia; LGE—late gadolinium enhancement; LVEF—left ventricular ejection fraction; LA—left atrial diameter; AD—aortic diameter; RV—right ventricle; TAPSE—tricuspid annular plane systolic excursion; MRI—magnetic resonance imaging; ABPM—ambulatory blood pressure monitoring; MAP—mean arterial pressure; PP—pulse pressure; TC—total cholesterol; HDL—high-density lipoprotein; TG—triglycerides.

## Data Availability

The data underlying this article cannot be shared publicly for the privacy of the individuals that participated in the study.
